# ASSESSMENT OF BONE MINERAL DENSITY BY DUAL X-RAY ABSORPTIOMETRY IN DERMATOLOGICAL PATIENTS TREATED BY CORTICOSTEROIDS

**DOI:** 10.4103/0019-5154.70669

**Published:** 2010

**Authors:** Dorria Salem, Soha Talaat, Mona R E Abdel-Halim, Kareem Mohammed Mohsen

**Affiliations:** *From the dept.of dermatology & radiodiagnosis, Cairo University, Cairo, Egypt*

**Keywords:** *Bone mineral density*, *corticosteroid induced*, *dual X-ray absorptiometry*, *dermatological patients*, *dexamethasone pulse therapy*, *osteoporosis*

## Abstract

**Background:**

Corticosteroids are mainstay of dermatological therapy and they are also a well known cause of osteoporosis. The objective of the present study was to find out the influence of the systemic intake of corticosteroids, either by the oral route or by IV pulse administration, on bone mineral density in dermatological patients using dual X-ray absorptiometry (DXA).

**Materials and Methods:**

This study was carried on 100 patients and 55 controls. The first group of patients included 55 patients undergoing long-term oral corticosteroid therapy daily and the second group included 45 patients who received IV dexamethasone pulse therapy. DXA was measured once for both the controls and patients in group 1. DXA was measured twice for patients in group 2, before starting pulse therapy (baseline DXA) and six months after regular treatment with pulse therapy (follow-up DXA).

**Results:**

The results show that significant reduction in BMD occurs in both groups, however, oral corticosteroids produce significantly more reduction in BMD in the lumbar spine. BMD was not found to be affected by the cumulative doses of corticosteroids, the duration of daily oral corticosteroid intake, or the number of IV dexamethasone pulses.

**Conclusion:**

Corticosteroid treatment causes significant BMD loss in patients treated by either route. Prophylactic treatment against osteoporosis is mandatory in patients receiving either form of corticosteroids.

## Introduction

Systemic corticosteroids are a mainstay of dermatological therapy because of their potent immunosuppressive and anti-inflammatory properties. They are used to treat patients with severe forms of various skin diseases such as pemphigus, bullous pemphigoid, erythema multiforme, dermatomyositis, systemic lupus erythematosus, and severe forms of dermatitis.[1]

Systemic intake of corticosteroids is a well known cause of osteoporosis[2] and corticosteroids affect the bone in many ways. They cause decreased bone formation and increased bone resorption. In addition, they decrease intestinal calcium absorption, increase urinary calcium excretion, and disturb vitamin D metabolism.[3] The incidence of corticosteroid-induced osteoporosis is approximately 50% in patients who have been treated for more than six months, and it has been estimated that over 34% of patients on long-term corticosteroids have had fractures.[4]

Unfortunately, osteoporosis is asymptomatic unless it results in a fracture, usually a vertebral compression fracture or a fracture of the wrist, hip, ribs, pelvis, or humerus. The middle and lower thoracic and upper lumbar regions are most frequently involved, which usually results in back pain. Fractures of the femoral neck and intertrochanteric region are the most devastating complications of osteoporosis. Hip fractures are associated with falls, occurring usually as a result of modest trauma. Secondary complications of hip fractures, such as pulmonary thromboembolism or nosocomial infections, carry a mortality rate of 15–20% in elderly patients.[5]

Over the past decade, dual X-ray absorptiometry (DXA) has established itself as the most widely used method to measure bone mineral density (BMD) due to its high precision, short scan times, and stable calibration in clinical use. DXA equipment allows scanning of the lumbar spine, proximal femur, and distal forearm. These are regarded as the most important measurement sites as they are frequently sites for fractures that cause substantial impairment of the quality of life and increased morbidity and mortality.[6] Assessment of BMD in these sites is an accurate marker for osteoporosis that helps to identify the risk for fracture.[7]

Most studies on the effects of corticosteroids on bone density and the efficacy of treatment regimens for preventing bone loss have been in patients with asthma and rheumatologic disease, such as rheumatoid arthritis and systemic lupus erythematosus. In both these diseases, osteoporosis can be a part of the disease process itself. At this time, there are no published studies on patients with bullous diseases who have no other systemic risk factors for osteoporosis and who require large doses of corticosteroids.[7]

The aim of this study was to examine the influence of systemic intake of corticosteroids, either by the daily oral method or IV pulse method, on bone mineral density in dermatological patients.

## Materials and Methods

### 

#### Subjects

This study was carried on 155 subjects, 100 patients suffering from a spectrum of dermatological diseases, and 55 volunteers who served as controls. The Ethics Committee approved the study and informed consent was obtained from the patients and the controls before measuring BMD.

#### Patients

### Group 1: Daily oral corticosteroids

This group included 55 patients (30 males and 25 females) aged 17–62 years. They had already received oral corticosteroids daily for at least six months. BMD was measured once for this group (cross-sectional evaluation).

### Group 2: Intravenous dexamethasone pulse

This group included 45 patients (14 males and 31 females) aged 17–65 years. These patients were to receive IV dexamethasone pulse therapy every 1–4 weeks with no daily oral corticosteroids in between. BMD was measured twice, before starting the first pulse (baseline DXA) and after six months (follow-up DXA) (prospective evaluation).

### Exclusion criteria

In order to avoid false results due to hypogonadism and postmenopausal osteoporosis, postmenopausal females and males above 65 years of age were excluded from the study.

### Data collection

Personal, clinical, and treatment data were collected from all patients. Clinical data included diagnosis and duration of illness. Data were collected for other risk factors for osteoporosis such as smoking, coffee, alcohol, hyperparathyroidism, hyperthyroidism, renal failure, ovariectomy, and history of fractures. The presence of any other disease was also recorded.

Treatment data included a detailed analysis of the corticosteroids taken by the patients. For patients in group 1, this included the type of the drug, the daily dose, and the duration of treatment. In group 2, patients were to receive 100 mg of dexamethasone daily for five days every 1–2 weeks until initial control of disease activity was achieved, then 100 mg of dexamethasone was to be given daily for five days every four weeks. Modified oral prednisone pulse therapy (60 mg/day for two consecutive days every week) was to be started when the initial disease activity was controlled and the patient was maintained on IV pulse dexamethasone every four weeks. The number of pulses received between baseline and follow-up DXA scans and the cumulative doses was calculated. All patients in group 2 would have received immunosuppressive therapy, such as azathioprine or cyclophosphamide, between the pulses. Most of the cases would be given pentoxifylline and colchicine. Past history of daily oral corticosteroids therapy as well as other medications taken by the patients in group 2 was recorded.

### Controls

Fifty-five volunteers served as controls, they included 22 males and 33 females aged 24–65 years.

#### Methods

### Measurement of BMD

All DXA measurements were done on Lunar Prodigy, GE medical system, USA. Before beginning work every day, the technologist performed a quality control check according to the manufacturer’s instructions.

BMD measurements were performed at three sites: the lumbar spine, the left hip, and the left forearm. During lumbar spine BMD measurement, the patient lay supine on the imaging table, the legs raised by support for the lower legs. In performing BMD measurement of the hip, the patient lay supine on the imaging table, their legs are flat on the table with feet strapped to a feet holder that position the measured leg at 30° inward rotation. For the left forearm BMD measurement, the patient sat beside the imaging table with the arm positioned on the imaging table supported with a positioning device.

The references used for BMD measurements are L2–L4 for the lumbar spine, the total femur for the proximal femur, and the one-third-radius for the forearm.

### Calculation of percentage change in group 2

The following formula was used for the calculation of the percentage change between baseline and follow-up data in the pulse group,:

$$\frac{\mathrm{Follow-up}\;\mathrm{BMD}\;\mathrm{measurement}\;—\;\mathrm{Baseline}\;\mathrm{BMD}\;\mathrm{measurement}}{\mathrm{Baseline}\;\mathrm{BMD}\;\mathrm{measurement}}\;\times \;100$$

The BMD change after the initiation of treatment must escape the least significant change value to be statistically significant, thus the change should exceed 2.8%. Changes in BMD of less than 2.8% could be due to inherent measurement errors.[8–11]

#### Statistical analysis

Data were encoded and entered using Microsoft Excel version 7 (Microsoft Corporation, NY, USA) and SPSS (Statistical Package for the Social Sciences; SPSS Inc., Chicago, IL, USA) statistical program. Data were statistically described in terms of range, mean, standard deviation (SD), median, frequencies (number of cases), and relative frequencies (percentages) whenever appropriate. Comparison of quantitative data between different groups in the present study was done using Student’s t-test for independent samples that were normally distributed and the Mann Whitney U test for independent samples when they were not normally distributed. Comparison between baseline and post-treatment data in the pulse group was done using Wilcoxon signed rank. Chi square^2^ test was used to compared data between the sexes. Yates correction was used instead when the expected frequency was less than 5. Correlation between different variables was done using Pearson’s moment linear correlation coefficient (r). A probability value (*P*) less than 0.05 was considered to be statistically significant.[12]

## Results

### 

#### Patients’ data

A variety of dermatological diseases was included in the study, pemphigus vulgaris was the most frequently observed (30 cases in group 1, 25 cases in group 2) [Table 1].

**Table 1:** Disease distribution in both groups of patients

Disease	Number of cases (group 1)	Number of cases (group 2)
Pemphigus vulgaris	30	25
Pemphigus erythematosus	5	-
Behçet’s disease	4	1
Mycosis fungoides	4	2
Systemic lupus erythematosus (Acute cutaneous LE)	3	2
Dermatomyositis	2	2
Pemphigoid	2	3
Psoriasis	2	2
Subacute lupus erythematosus	1	1
Vasculitis	1	5
Vitiligo	1	1
Erythema multiforme	-	1
Herpes gestation	-	1

### Group 1: Daily oral corticosteroids

This group included 55 patients: 25 females and 30 males aged 17–62 years (median: 45 years). The cumulative dose of corticosteroids taken ranged from 1.8 to 72.4 g of prednisone (mean: 24.79 ± 18.47 g). The duration of corticosteroids intake ranged from six to 96 months (median: 24 months).

### Group 2: Intravenous dexamethasone pulse

This group included 45 patients: 31 females and 14 males aged 17–65 years (median: 38 years). The cumulative dose taken ranged from 2.5 to 10 g (mean: 5.43 ± 2.08 g) and the duration was 5–9 months (mean: 7.53 ± 2.04). The number of pulses taken was 6–20 pulses (median: 10). In this group, 12 patients received oral corticosteroids daily prior to presentation, but they were stopped for more than one month before starting the pulse therapy. Exact doses of oral corticosteroids taken in the past could not be evaluated.

Patients in both groups 1 and 2 were age-matched (*P* = 0.469), however, there was a significant difference between both groups (*P* = 0.018) with respect to sex distribution, with more females being present in group 2.

### Controls’ data

Fifty-five volunteers served as controls; they included 33 females and 22 males aged 24–65 years (median: 46 years) Patients in both groups were both age- and sex-matched with the controls (*P* > 0.05).

### Results of BMD measurements

### I. Within group 1:

### Comparisons of BMD and T-scores between patients and controls:

**Figure 1:**
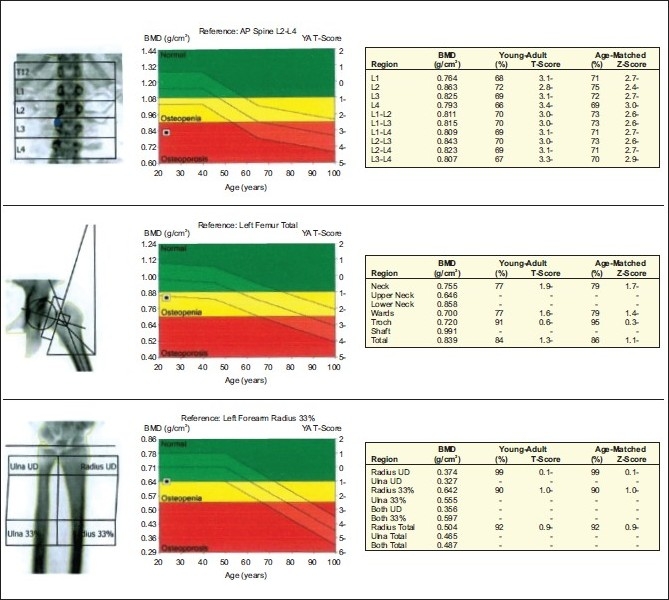
DEXA of a female patient, 23 years old, with vasculitis, on daily oral corticosteroids for one year (18.3 g cumulative dose) showing osteoporosis in the lumbar spine and osteopenia in the left hip

**Figure 2:**
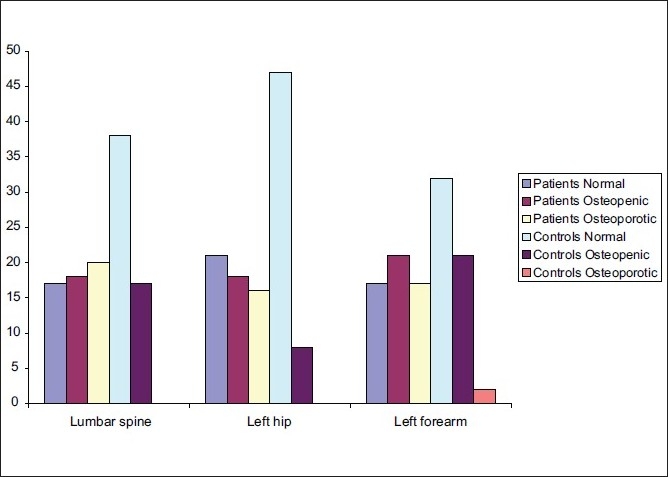
T-scores in patients (group 1) and controls

Compared to the controls [Figure 1], BMD measurements in the patients were significantly lower in the lumbar spine and left hip, but no significant difference was found in the left forearm [Table 2]. T-score results are shown in Table 3 and Figure 2 as normal BMD, osteopenia, or osteoporosis.

**Table 2:** Comparison of BMD between patients in group 1 and controls

	Lumbar spine BMD (g/cm^2^)	Left hip BMD (g/cm^2^)	Left forearm BMD (g/cm^2^)
Patients (n = 55)			
Maximum	1.429	1.181	0.817
Minimum	0.661	0.426	0.506
Mean	0.97	0.86	0.66
SD	0.18	0.18	0.08
Median	0.976	0.889	0.678
Controls (n = 55)			
Maximum	1.550	1.425	0.852
Minimum	0.975	0.719	0.503
Mean	1.17	1.01	0.67
SD	0.13	0.13	0.06
Median	1.146	1.022	0.672
Analysis			
*P* value	< 0.001	0.016	0.486

**Table 3:** T-score results shown as normal BMD, osteopenia, and osteoporosis in the patients in group 1 and controls

	Lumbar spine	Left hip	Left forearm
Patients (n = 55)			
Normal	17 (30.9)	21 (38.2)	17 (30.9)
Osteopenic	18 (32.7)	18 (32.7)	21 (38.2)
Osteoporotic	20 (36.4)	16 (29.1)	17 (30.9)
Controls (n = 55)			
Normal	38 (69.1)	47 (85.5)	32 (58.2)
Osteopenic	17 (30.9)	8 (14.5)	21 (38.2)
Osteoporotic	0 (0)	0 (0)	2 (3.6)
Analysis			
*P* value	< 0.001	< 0.001	< 0.001

Figures in parenthesis are in percentage

### Comparison of BMD between males and females in the patients’ group:

When comparing male patients to female patients, no significant difference was found in the BMD of the lumbar spine (*P* = 0.636), left hip (*P* = 0.852), or the left forearm (*P* = 0.139).

### Studying correlation between BMD and age of patients:

There was a significant decrease in BMD in the lumbar spine (r = -0.4414, *P* = 0.001) and left hip (r = -0.3878, *P* = 0.004) with increasing age of patients, but no significant difference was found in the left forearm (r = -0.1195, *P* = 0.399)

### Studying correlation between BMD and both the cumulative dose of corticosteroids and duration of intake:

Multiple correlation analyses showed that BMD decreased significantly when correlated with the cumulative dose in the left hip (r = -0.3296, *P* = 0.016) for the patients, with no significant correlation in the lumbar spine (r = -0.1838, *P* = 0.179) and left forearm (r = -0.0614, *P* = 0.665). No significant correlation was found between BMD and the duration of corticosteroid intake.

### II. Within group 2:

### Comparison of BMD between patients (baseline) and controls:

**Figure 3:**
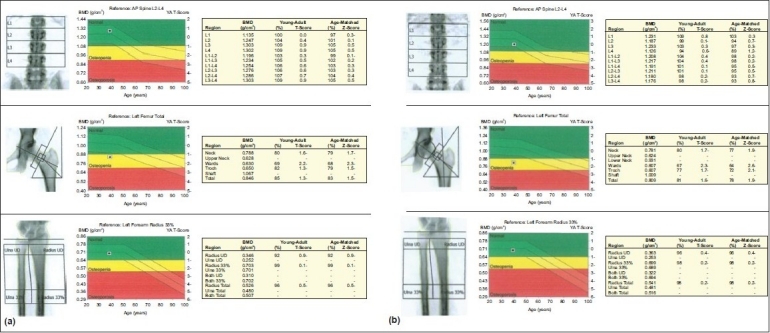
Baseline (Figure 3a) and follow-up DEXA (Figure 3b) of a female patient, 38 years old, with pemphigus vulgaris who received 15 pulses of IV dexamethasone over nine months with a cumulative dose of 7.5 g. Both figures show osteopenia in the left hip only

BMD was significantly lower in the patients than in controls in the lumbar spine (1.10 *vs* 1.17 g/cm^2^, *P* = 0.007) and the left hip (0.94 *vs* 1.01 g/cm^2^, *P* = 0.002). No significant difference was found in the left forearm (0.68 *vs* 0.67 g/cm^2^, *P* = 0.434) [Figure 3].

### Comparison of BMD and T-scores between baseline and follow-up DXA scans

**Figure 4:**
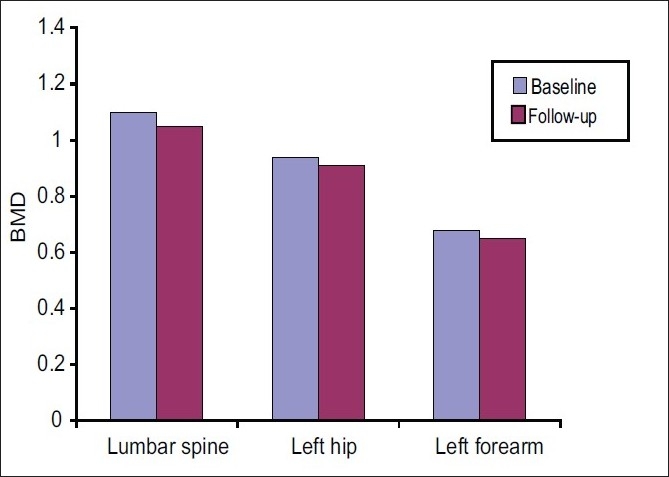
Comparison of BMD between baseline and follow-up DXA scans (group 2)

**Figure 5:**
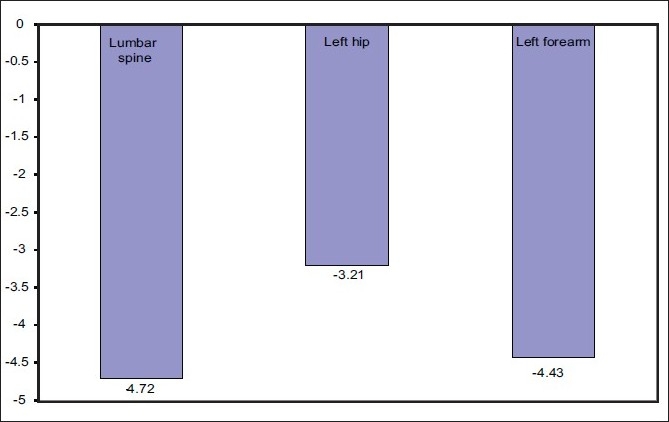
Mean percentage change of BMD between baseline and follow-up DXA scans (group 2)

BMD decreased significantly in the lumbar spine, left hip, and left forearm [Table 4 and Figure 4]. The mean percentage change of BMD was -4.72, -3.21, and -4.43 in the lumbar spine, left hip, and left forearm respectively [Figure 5]. T-score results are shown in Table 5 as normal BMD, osteopenia, or osteoporosis.

**Table 4:** BMD in the patients (group 2) before and after treatment with IV dexamethasone pulse

	Lumbar spine BMD (g/cm^2^)	Left hip BMD (g/cm^2^)	Left forearm BMD (g/cm^2^)
Baseline			
Maximum	1.303	1.321	0.831
Minimum	0.839	0.783	0.529
Mean	1.10	0.94	0.68
SD	0.12	0.10	0.07
Median	1.088	0.907	0.675
Follow-up			
Maximum	1.287	1.208	0.795
Minimum	0.769	0.664	0.464
Mean	1.05	0.91	0.65
SD	0.14	0.11	0.08
Median	1.065	0.893	0.665
Analysis			
*P* value	< 0.001	0.007	< 0.001

**Table 5:** T-score changes in group 2 between baseline and follow-up DXA scans

	Lumbar spine	Left hip	Left forearm
Baseline			
Normal	28 (62.2)	28 (62.2)	20 (44.4)
Osteopenic	13 (28.9)	15 (33.3)	17 (37.8)
Osteoporotic	4 (8.9)	2 (4.4)	8 (17.8)
Follow-up			
Normal	22 (48.9)	22 (48.9)	23 (51.1)
Osteopenic	19 (42.2)	19 (42.2)	12 (26.7)
Osteoporotic	4 (8.9)	4 (8.9)	10 (22.2)
Analysis			
*P* value	0.338	0.177	0.893

Figures in parenthesis are in percentage

### Comparison of BMD between males and females in both baseline and follow-up DXA scans

BMD values were significantly lower in males compared to the BMD values in both scans in the lumbar spine in females (*P* = 0.021). There was no significant difference between both sexes in the baseline scan of the left hip (*P* = 0.056) and in both scans of the forearm (*P* = 0.559). BMD was significantly lower in males in the left hip in the follow-up scan.

### Studying correlations between BMD and age in group 2 patients

Baseline BMD values showed a significant inverse correlation with age only in the left hip (r = -0.3434, *P* = 0.021), with no significant correlation in the lumbar spine (r = -0.2231, *P* = 0.141) and left forearm (r = -0.0690, *P* = 0.652). There was a significant decrease in the follow-up BMD values in the lumbar spine (r = -0.3358, *P* = 0.024), left hip (r = -0.3488, *P* = 0.019), and left forearm (r = -0.3653, *P* = 0.014) with increasing age.

### Studying correlations between BMD and the cumulative doses of dexamethasone as well as the number of pulses in group 2 patients

Multiple correlations analyses showed that there were no significant correlations between the follow-up BMD and the cumulative dose and number of pulses in the lumbar spine, left hip, and left forearm for the patients.

### III. Comparison of BMD between both groups

**Figure 6:**
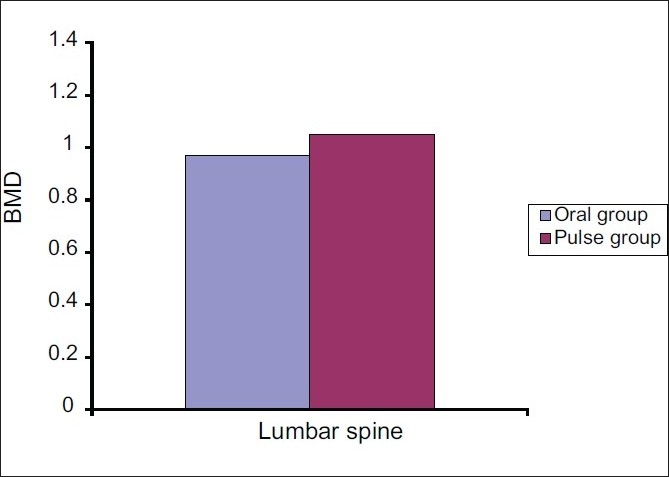
Difference in BMD between patients in group 1 (daily oral corticosteroids) and patients in group 2 (pulse group) in the lumbar spine (*P* < 0.022)

Comparison between the patients treated with oral corticosteroids and those treated by IV pulse corticosteroids showed that the BMD was significantly lower in the lumbar spine (*P* = 0.022) in patients treated with oral corticosteroids, whereas no significant difference was found in the left hip (*P* = 0.145) or the left forearm (*P* = 0.571) [Table 6 and Figure 6].

**Table 6:** Comparison of BMD between both groups of patients

	Lumbar spine BMD (g/cm^2^)	Left hip BMD (g/cm^2^)	Left forearm BMD (g/cm^2^)
Oral group (*n* = 55)			
Maximum	1.429	1.181	0.817
Minimum	0.661	0.426	0.506
Mean	0.97	0.86	0.66
SD	0.18	0.18	0.08
Median	0.976	0.889	0.678
Pulse group (follow-up measurement) (*n* = 45)			
Maximum	1.287	1.208	0.795
Minimum	0.769	0.664	0.464
Mean	1.05	0.91	0.65
SD	0.14	0.11	0.08
Median	1.065	0.893	0.665
Analysis			
*P*	0.022	0.145	0.571

## Discussion

One hundred dermatological patients requiring treatment by systemic corticosteroids were evaluated by DXA in this study. They included two groups: patients receiving longterm oral corticosteroids daily and patients receiving IV dexamethasone pulse.

In agreement with studies in rheumatological patients with systemic lupus erythematosus (SLE)[13,14] and in patients with chronic obstructive pulmonary disease, this study showed that BMD was significantly reduced in both the lumbar spine (*P* < 0.001) and the left hip (*P* = 0.016) in dermatological patients receiving long-term oral corticosteroid therapy when compared to controls.

The difference in BMD between the patients and the controls was obviously more significant in the lumbar spine than in the left hip and the forearm (*P* < 0.001 and *P* = 0.016 respectively). It appears that the more metabolically active trabecular sites (lumbar spine) are more prone to corticosteroid-induced osteoporosis than the cortical sites (hip).[15,16]

Although weak, a significant inverse correlation was found between BMD and the cumulative dose of oral corticosteroid in the left hip (r = -0.3296, *P* = 0.016); no similar correlation was found in the lumbar spine. It is postulated that corticosteroid-induced bone loss of cortical bones increases with increased cumulative dose in contrast to the more metabolically active trabecular bones. On the other hand, the duration of intake of oral steroids did not show any correlation with BMD in this study. Similar to the results of this study,[13] found no correlation between BMD and the average daily dose, cumulative dose, or duration of corticosteroid intake. However, a meta-analysis of 89 studies examining the effects of corticosteroids intake on BMD and fracture risk, reported that cumulative doses are strongly correlated with loss of BMD and higher daily doses are associated with increased risk of fractures.[17]

Within group 2, baseline BMD was significantly lower in patients than in controls, both in the lumbar spine and the left hip. No significant difference was found in the forearm. The main dermatological disease in this group was pemphigus vulgaris (PV), a severe form of an immunobullous disorder. Immunobullous disorders do not directly affect BMD by themselves.[7] However, most PV cases have painful oral mucosal erosions that make it difficult for patients to eat or drink adequately and may be a cause of malnutrition, leading to deficiency of calcium, vitamin D, and other minerals needed for bone metabolism.[18,19] Furthermore, 12 out of 45 (26.7%) of patients in this group reported receiving oral corticosteroids daily prior to presentation, although the exact dose could not be recalled by the patients. This may contribute to the reduction in BMD seen in this group in the baseline DXA measurements.

BMD decreased by 4.72, 3.21, and 4.43% in the lumbar spine, left hip, and left forearm respectively between the baseline and the follow-up DXA measurements in group 2.

Patients in group 2 also received immunosuppressive drugs such as azathioprine and cyclophosphamide. Although immunosuppressive drugs are reported to cause bone mineral loss in patients after organ transplantation, no such relation was reported in patients with autoimmune diseases requiring treatment with immunosuppressive drugs. This can be attributed to the different doses and regimens of treatment by immunosuppressive drugs in these patients—autoimmune patients require lower doses.[20–22] Additional effects of weekly pulses of oral 60 mg of prednisone after control of the initial disease activity cannot be excluded in this group of patients. Others have reported that weekly pulses of oral 60 mg prednisone produce osteopenia and not osteoporosis.

It appears that IV dexamethasone pulse therapy affected BMD in the lumbar spine more than in the left hip (4.72% change and 3.21% change respectively). BMD values in both sites were significantly lower than those of controls. However, an important finding in this study was that IV dexamethasone pulse therapy did cause a significant reduction in BMD in the left forearm (4.43% change, *P* < 0.001). This reduction was less than that observed in the lumbar spine, but was obviously more than that of the left hip. However, the mean BMD in the forearm in the followup DXA still did not differ significantly from the controls. It appears that the administration of high dexamethasone doses in pulse forms affects trabecular bone (more metabolically active) more than it affects cortical bone. However, these high doses may represent an impending risk for the development of osteoporosis in cortical sites such as the forearm also.

Several previous studies have concluded that bone metabolism is not seriously affected during corticosteroid pulse therapy and that the effect of methylprednisolone or dexamethasone pulse treatment on bone may be assumed to be relatively mild. Yet these studies did not measure the BMD by any densiometric technique. They relied on different markers for assessment of bone and calcium metabolism such as serum calcium, parathyroid hormone, vitamin D, alkaline phosphatase, and osteocalcin in addition to urinary excretion of calcium, hydroxyproline, deoxypyridinoline, and pyridinoline.[23–25]

On the other hand, a recent study measured the BMD using DXA at baseline and after 5.7 months in the lumbar spine and hip for 38 patients with various rheumatic disorders requiring IV methylprednisolone pulse. During the study, the patients received a mean cumulative dose of 3.0 g of methylprednisolone. A high rate of bone loss was detected and at the end of the study, the mean BMD was reduced by 1.0% in the lumbar spine and 1.1% in the hip.[2] Literature reviews did not reveal any similar studies using IV dexamethasone pulse therapy.

No significant correlation was found between both the cumulative dose and the number of pulses of dexamethasone and the BMD in the lumbar spine, left hip, and left forearm. However, BMD was found to negatively correlate with the cumulative dose of methylprednisolone in a study by Haugeberg and colleagues.[2] It is known that dexamethasone has a longer duration of action (36–54 h) than methylprednisolone (24–36 h).[26] Studies comparing the effects of methylprednisolone and dexamethasone on BMD are needed.

No statistically significant difference was found between males and females receiving oral corticosteroids daily with respect to the BMD in the lumbar spine, left hip, and left forearm. It appears that such oral corticosteroid-induced osteoporosis is not affected by gender. No studies have compared BMD in male and female patients suffering from corticosteroid-induced osteoporosis to date. Most of the earlier studies were done on female patients with rheumatoid arthritis and SLE. On the other hand, BMD values were significantly lower in males at the baseline DXA measurements (lumbar spine) and the follow-up DXA measurements (lumbar spine and left hip) within the pulse group. This can be explained by the higher mean age of the males in this study (49.78 years) as compared to the females (33.25 years). Osteoporosis is underdiagnosed in older men despite its significant association with disability and death. A population-based, multicenter osteoporosis study (CaMos) found that the prevalence of vertebral deformities in subjects over the age of 50 years was 21.5% among men and 23.5% among women.[27]

Corticosteroid-induced reductions in BMD appear to increase with increasing age of patients in both groups. Older patients are more prone to the risk of corticosteroidinduced osteoporosis.

BMD values in the lumbar spine were significantly lower in the daily oral corticosteroids group than in the IV dexamethasone pulse group. This may suggest that the latter therapy may have a less harmful effect on the lumbar spine (a metabolically active site).

Although the pulse group patients showed a significant reduction in BMD, the change was not large enough to cause the patients to change from one diagnostic group to the other based on T-score values (*i.e*., no significant number of patients changed from having normal BMD to being osteopenic or osteoporotic). On the other hand, a significant number of oral group patients were diagnosed as having osteopenia and osteoporosis. These findings also show that the IV corticosteroid pulses cause less significant osteoporosis than the oral route.

## Conclusion

Both routes of corticosteroid treatment cause significant BMD loss. However, pulse therapy is less harmful to the lumbar spine. No significant correlation was found between the cumulative dose and the duration of oral corticosteroids/number of pulses and the BMD, except for a cumulative dose-dependent effect in the left hip. Prophylactic treatment against osteoporosis is mandatory for patients receiving corticosteroids orally or in the form of pulse therapy.

## Recommendations

Further studies are needed which would measure the BMD before and after daily oral corticosteroid intake in order to compare the extent of BMD loss produced by oral corticosteroids and that produced by IV pulse therapy. Studies comparing the effects of both methylprednisolone and dexamethasone in reducing BMD are also required, keeping in mind the difference in the duration of biological activity of these two forms.
